# Pediatric Death Due to Myocarditis After Exposure to Cannabis

**DOI:** 10.5811/cpcem.2017.1.33240

**Published:** 2017-03-16

**Authors:** Thomas M. Nappe, Christopher O. Hoyte

**Affiliations:** * Denver Health and Hospital Authority, Rocky Mountain Poison and Drug Center, Denver, Colorado; † University of Colorodo School of Medicine at Anschutz Medical Center, Department of Emergency Medicine, Aurora, Colorado

## Abstract

Since marijuana legalization, pediatric exposures to cannabis have increased.^1^ To date, pediatric deaths from cannabis exposure have not been reported. The authors report an 11-month-old male who, following cannabis exposure, presented with central nervous system depression after seizure, and progressed to cardiac arrest and died. Myocarditis was diagnosed post-mortem and cannabis exposure was confirmed. Given the temporal relationship of these two rare occurrences – cannabis exposure and sudden death secondary to myocarditis in an 11-month-old – as well as histological consistency with drug-induced myocarditis without confirmed alternate causes, and prior reported cases of cannabis-associated myocarditis, a possible relationship exists between cannabis exposure in this child and myocarditis leading to death. In areas where marijuana is commercially available or decriminalized, the authors urge clinicians to preventively counsel parents and to include cannabis exposure in the differential diagnosis of patients presenting with myocarditis.

## INTRODUCTION

Since marijuana legalization, pediatric exposures to cannabis have increased, resulting in increased pediatric emergency department (ED) visits.^1^ Neurologic toxicity is most common after pediatric exposure; however, gastrointestinal and cardiopulmonary toxicity are reported.^1^ According to a retrospective review of 986 pediatric cannabis ingestions from 2005 to 2011, pediatric exposure has been specifically linked to a multitude of symptoms including, but not limited to, drowsiness, lethargy, irritability, seizures, nausea and vomiting, respiratory depression, bradycardia and hypotension.^1^ Prognosis is often reassuring.^1^ Specific myocardial complications related to cannabis toxicity that are well documented in adolescence through older adulthood include acute coronary syndrome, cardiomyopathy, myocarditis, pericarditis, dysrhythmias and cardiac arrest.^2^–^4^ To date, there are no reported pediatric deaths from myocarditis after confirmed, recent cannabis exposure. The authors report an 11-month-old male who, following cannabis exposure, presented in cardiac arrest after seizure and died. Myocarditis was diagnosed post-mortem and cannabis exposure was confirmed. Analyses of serum cannabis metabolites, post-mortem infectious testing, cardiac histopathology, as well as clinical course, support a potential link between the cannabis exposure and myocarditis that would justify preventive parental counseling and consideration of urine drug screening in this reported setting.

## CASE REPORT

**Image 1:**
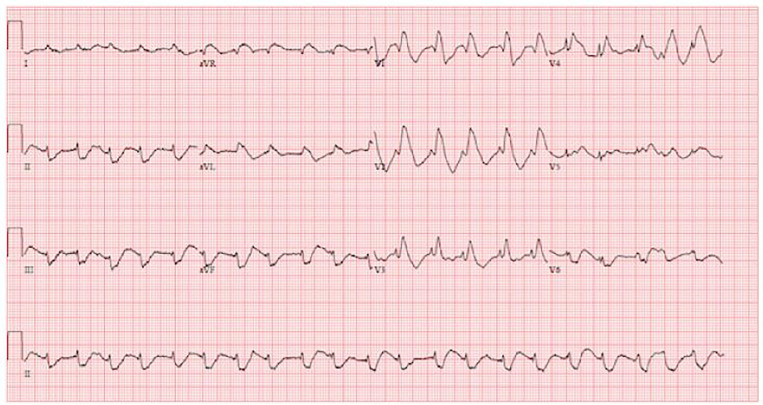
Initial electrocardiogram demonstrating wide-complex tachycardia.

**Image 2:**
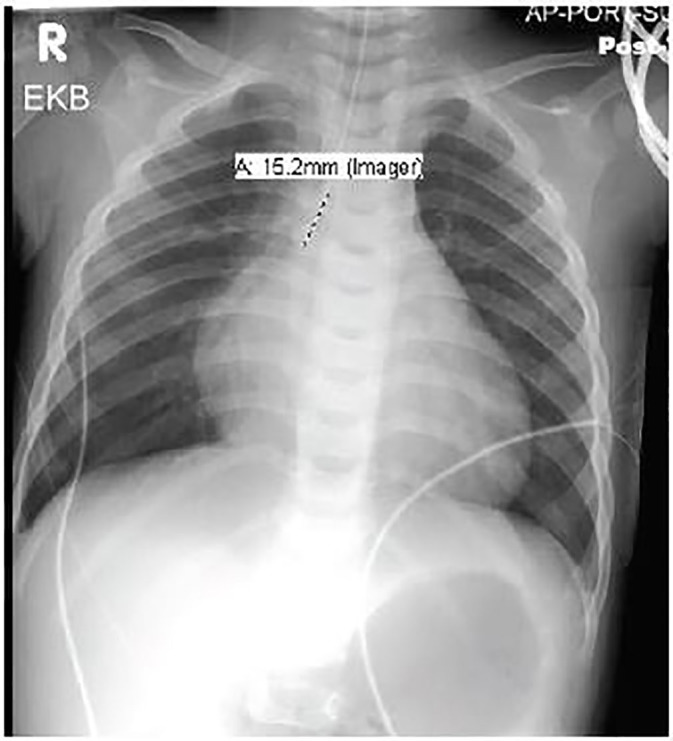
Post-intubation chest radiograph. Measurement indicates distance of endotracheal tube tip above carina.

**Image 3:**
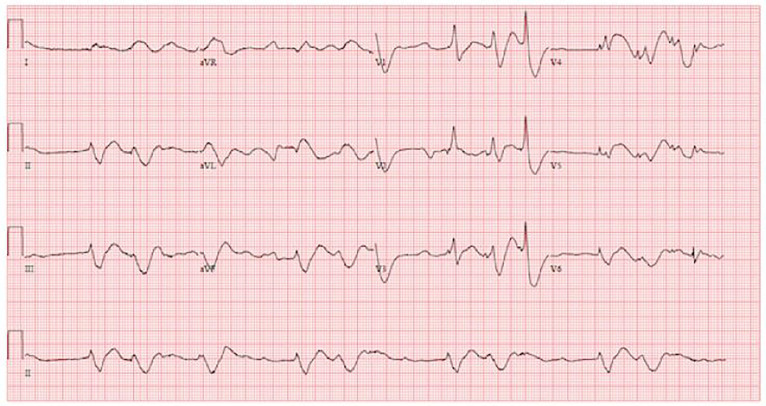
Repeat electrocardiogram showing disorganized rhythm, peri-arrest.

An 11-month-old male with no known past medical history presented to the ED with central nervous system (CNS) depression and then went into cardiac arrest. The patient was lethargic for two hours after awakening that morning and then had a seizure. During the prior 24–48 hours, he was irritable with decreased activity and was later retching. He was noted to be healthy before developing these symptoms. Upon arrival in the ED, he was unresponsive with no gag reflex. Vital signs were temperature 36.1° Celsius, heart rate 156 beats per minute, respiratory rate 8 breaths per minute, oxygen saturation 80% on room air. Physical exam revealed a well-nourished, 20.5 lb., 11-month-old male, with normal development, no trauma, normal oropharynx, normal tympanic membranes, no lymphadenopathy, tachycardia, clear lungs, normal abdomen and Glasgow Coma Scale rating of 4. He was intubated for significant CNS depression and required no medications for induction or paralysis. Post-intubation chest radiograph is shown in Image 2. He subsequently became bradycardic with a heart rate in the 40s with a wide complex rhythm. Initial electrocardiogram (ECG) was performed and is shown in Image 1. He then became pulseless, and cardiopulmonary resuscitation was initiated. Laboratory analysis revealed sodium 136 mmol/L, potassium 7.7 mmol/L, chloride 115 mmol/L, bicarbonate 8.0 mmol/L, blood urea nitrogen 24 mg/dL, creatinine 0.9 mg/dL, and glucose 175 mg/dL Venous blood gas pH was 6.77. An ECG was repeated (Image 3). He received intravenous fluid resuscitation, sodium bicarbonate infusion, calcium chloride, insulin, glucose, ceftriaxone and four doses of epinephrine. Resuscitation continued for approximately one hour but the patient ultimately died.

Further laboratory findings in the ED included a complete blood count (CBC) with differential, liver function tests (LFTs), one blood culture and toxicology screen. CBC demonstrated white blood cell count 13.8 K/mcL with absolute neutrophil count of 2.5 K/mcL and absolute lymphocyte count of 10.7 K/mcL, hemoglobin 10.0 gm/dL, hematocrit 34.7%, and platelet count 321 K/mcL. LFTs showed total bilirubin 0.6 mg/dL, aspartate aminotransferase 77 IU/L, and alanine transferase 97 IU/U. A single blood culture from the right external jugular vein revealed aerobic gram-positive rods that were reported two days later as Bacillus species (not *Bacillus anthracis*). Toxicology screening revealed urine enzyme-linked immunosorbent assay positive for tetrahydrocannabinol-carboxylic acid (THC-COOH) and undetectable serum acetaminophen and salicylate concentrations. Route and timing of exposure to cannabis were unknown.

Autopsy revealed a non-dilated heart with normal coronary arteries. Microscopic examination showed a severe, diffuse, primarily lymphocytic myocarditis, with a mixed cellular infiltrate in some areas consisting of histiocytes, plasma cells, and eosinophils. Myocyte necrosis was also observed. There was no evidence of concomitant bacterial or viral infection based on post-mortem cultures obtained from cardiac and peripheral blood, lung pleura, nasopharynx and cerebrospinal fluid. Post-mortem cardiac blood analysis confirmed the presence of Δ-9-carboxy-tetrahydrocannabinol (Δ-9-carboxy-THC) at a concentration of 7.8 ng/mL. Additional history disclosed an unstable motel-living situation and parental admission of drug possession, including cannabis.

## DISCUSSION

As of this writing, this is the first reported pediatric death associated with cannabis exposure. Given the existing relationship between cannabis and cardiovascular (CV) toxicity, as well as the temporal progression of events, post-mortem analysis, and previously reported cases of cannabis-induced myocarditis, the authors propose a relationship between cannabis exposure in this patient and myocarditis, leading to cardiac arrest and ultimately death. This occurrence should justify consideration of urine drug screening for cannabis in pediatric patients presenting with myocarditis of unknown etiology in areas where cannabis is widely used. In addition, parents should be counseled regarding measures to prevent such exposures.

The progressive clinical presentation of this patient during the prior 24–48 hours, including symptoms of somnolence, lethargy, irritability, nausea, seizure and respiratory depression are consistent with previously documented, known complications of recent cannabis exposure in the pediatric population.^1^ It is well known that common CV effects of cannabis exposure include tachycardia and decreased vascular resistance with acute use and bradycardia in more chronic use.^2^,^5^–^7^. These effects are believed to be multifactorial, and evidence suggests that cannabinoid effect on the autonomic nervous system, peripheral vasculature, cardiac microvasculature, and myocardial tissue and Purkinje fibers are all likely contributory.^2^ The pathogenesis of myocarditis is not fully understood. In general, myocarditis results from direct damage to myocytes from an offending agent such as a virus, or in this case, potentially a toxin. The resulting cellular injury leads to a local inflammatory response. Destruction of cardiac tissue may result in myocyte necrosis and arrhythmogenic activity, or cellular remodeling in chronic myocarditis.^8^,^9^

Autopsy findings in this patient were consistent with noninfectious myocarditis as a cause of death. The histological findings of myocyte necrosis with mature lymphocytic mixed cellular infiltrate are consistent with drug-induced, toxic myocarditis.^10^ The presence of THC metabolites in the patient’s urine and serum, most likely secondary to ingestion, is the only uncovered risk factor in the etiology for his myocarditis. This is highly unlikely attributable to passive exposure. 11,12

It is difficult to extrapolate a specific time of cannabis ingestion given the unknown dose of THC, the individual variability of metabolism and excretion, as well as the lack of data on this topic in the pediatric population and post-mortem redistribution (PMR) kinetics. However, the THC metabolite detected in the patient’s blood, Δ-9-carboxy-THC, is known to peak in less than six hours and be detectable for at least a day, while the parent compound, tetrahydrocannabinol (THC), is expected to rapidly metabolize and distribute much more quickly, being potentially undetectable six hours after exposure in an infrequent user.^13^ The parent compound was below threshold for detection in this patient’s blood. In addition, if cannabis ingestion occurred the day of presentation, it would have been more likely that THC would have been detected with its metabolite after PMR.^14^,^15^ Given this information, the authors deduce that cannabis consumption occurred within the recent two to six days, assuming this was a single, acute high-potency ingestion. This time frame would overlap with the patient’s symptomatology and allow time for the development of myocarditis, thus supporting cannabis as the etiology.

The link between cannabis use and myocarditis has been documented in multiple teenagers and young adults.^16^–^18^ In 2008 Leontiadis reported a 16-year-old with severe heart failure requiring a left ventricular assist device, associated with biopsy-diagnosed myocarditis.^16^ The authors attributed the heart failure to cannabis use of unknown chronicity.^16^ In 2014 Rodríguez-Castro reported a 29-year-old male who had two episodes of myopericarditis several months apart.^17^ Each episode occurred within two days of smoking cannabis. 18 In 2016, Tournebize reported a 15-year-old male diagnosed with myocarditis, clinically and by cardiac magnetic resonance imaging, after initiating regular cannabis use eight months earlier.^18^ There were no other causes for myocarditis, including infectious, uncovered by these authors, and no adulterants were identified in these patients’ consumed marijuana.^16^–^18^ Unlike our patient, all three of these previously reported patients recovered.

In the age of legalized marijuana, children are at increased risk of exposure, mainly through ingestion of food products, or “edibles.”^19^ These products are attractive in appearance and have very high concentrations of THC, which can make small exposures exceptionally more toxic in small children. 19,20

Limitations in this report include the case study design, the limitations on interpreting an exact time, dose and route of cannabis exposure, the specificity of histopathology being used to classify etiology of myocarditis, and inconsistent blood culture results. The inconsistency in blood culture results also raises concern of a contributing bacterial etiology in the development of myocarditis, lending to the possibility that cannabis may have potentially induced the fatal symptomatology in an already-developing silent myocarditis. However, due to high contaminant rates associated with bacillus species and negative subsequent blood cultures, the authors believe this was more likely a contaminant.^21^ In addition, the patient had no source of infection on exam or recent history and was afebrile without leukocytosis.^22^ All of his subsequent cultures from multiple sites were negative.

## CONCLUSION

Of all the previously reported cases of cannabis-induced myocarditis, patients were previously healthy and no evidence was found for other etiologies. All of the prior reported cases were associated with full recovery. In this reported case, however, the patient died after myocarditis-associated cardiac arrest. Given two rare occurrences with a clear temporal relationship – the recent exposure to cannabis and the myocarditis-associated cardiac arrest – we believe there exists a plausible relationship that justifies further research into cannabis-associated cardiotoxicity and related practice adjustments. In states where cannabis is legalized, it is important that physicians not only counsel parents on preventing exposure to cannabis, but to also consider cannabis toxicity in unexplained pediatric myocarditis and cardiac deaths as a basis for urine drug screening in this setting.
